# Ortho­rhom­bic modification of bis­[4-(3-pyridyl­methyl­idene­amino)­phen­yl]methane

**DOI:** 10.1107/S1600536810032034

**Published:** 2010-08-18

**Authors:** Kiramat Shah, Muhammad Raza Shah, Islam Ullah Khan, Seik Weng Ng

**Affiliations:** a H.E.J. Research Institute of Chemistry, International Center for Chemical and Biological Sciences, University of Karachi, Karachi 75270, Pakistan; b Department of Chemistry, Government College University, 54000 Lahore, Pakistan; c Department of Chemistry, University of Malaya, 50603 Kuala Lumpur, Malaysia

## Abstract

The title compound, C_25_H_20_N_4_, is a disubstituted methane derivative having two pyridyl­methyl­ene­amino­phenyl arms, one of which is essentially rigid as all atoms lie on a plane (r.m.s. deviation = 0.074 Å), whereas the other is twisted [dihedral angle between the phenyl­ene and pyridyl rings = 51.1 (4)°]. The angle at the methyl­ene C atom is 113.2 (2)°.

## Related literature

For the monoclinic modification, see: Wang *et al.* (2005 ▶).
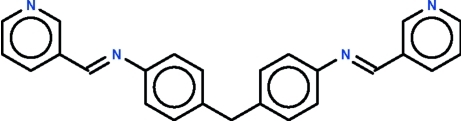


## Experimental

### 

#### Crystal data

C_25_H_20_N_4_
*M* *_r_* = 376.45
Orthorhombic, 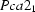

*a* = 11.4213 (4) Å
*b* = 4.6181 (2) Å
*c* = 37.3048 (15) Å
*V* = 1967.63 (14) Å^3^
*Z* = 4
Mo *K*α radiation
μ = 0.08 mm^−1^
*T* = 293 K
0.35 × 0.25 × 0.15 mm

#### Data collection

Bruker Kappa APEXII diffractometer
9233 measured reflections
1761 independent reflections
1450 reflections with *I* > 2σ(*I*)
*R*_int_ = 0.039

#### Refinement

*R*[*F* ^2^ > 2σ(*F* ^2^)] = 0.038
*wR*(*F* ^2^) = 0.109
*S* = 0.92
1761 reflections
262 parameters
1 restraint
H-atom parameters constrained
Δρ_max_ = 0.11 e Å^−3^
Δρ_min_ = −0.11 e Å^−3^

### 

Data collection: *APEX2* (Bruker, 2009 ▶); cell refinement: *SAINT* (Bruker, 2009 ▶); data reduction: *SAINT*; program(s) used to solve structure: *SHELXS97* (Sheldrick, 2008 ▶); program(s) used to refine structure: *SHELXL97* (Sheldrick, 2008 ▶); molecular graphics: *X-SEED* (Barbour, 2001 ▶); software used to prepare material for publication: *publCIF* (Westrip, 2010 ▶).

## Supplementary Material

Crystal structure: contains datablocks global, I. DOI: 10.1107/S1600536810032034/nk2051sup1.cif

Structure factors: contains datablocks I. DOI: 10.1107/S1600536810032034/nk2051Isup2.hkl

Additional supplementary materials:  crystallographic information; 3D view; checkCIF report

## Figures and Tables

**Fig. 1.**
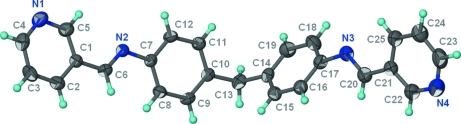
Displacement ellipsoid plot of C25H20N4 at the 50% probability level; hydrogen atoms are drawn as spheres of arbitrary radius.

## supplementary crystallographic information

### Comment

We have synthesized bis[4-(3-pyridylmethyleneamino)phenyl]methane (Scheme I) for use in cocrystallization studies. The compound has previously been reported to belong to the non-centric *Pc* space group [*a* = 18.751 (5), *b* = 4.641 (2), *c* = 11.387 (1) Å, *β* = 98.39 (2) °] (Wang *et al.*, 2005). The atoms of one of the two pyridylmethyleneaminophenylarms of the approximately *V*-shaped molecule all lie on a plane; for the other arm, the two aromatic rings are aligned at 53.9 (2) °]. The present orthorhombic modification (Fig. 1) has one axis that is doubled [*c* = 37.3048 (15), *b* = 4.6181 (2), *a* = 11.4213 (4) Å]. The principal features are similar to those of the reported structure.

### Experimental

The compound was synthesized from the condensation of 4,4'-diaminodiphenylmethane and 3-pyridinecarboxaldehyde in ethanol (Wang *et al.*, 2005), and crystals were obtained upon recrystallization from ethyl acetate.

### Refinement

Carbon-bound H-atoms were placed in calculated positions (C–H 0.93 to 0.97 Å) and were included in the refinement in the riding model approximation, with *U*(H) set to 1.2*U*(C). Friedel pairs (1535) were merged.

### Crystal data

**Table:**

C_25_H_20_N_4_	*F*(000) = 792
*M**_r_* = 376.45	*D*_x_ = 1.271 Mg m^−^^3^
Orthorhombic, *P**c**a*2_1_	Mo *K*α radiation, λ = 0.71073 Å
Hall symbol: P 2c -2ac	Cell parameters from 1793 reflections
*a* = 11.4213 (4) Å	θ = 3.3–20.6°
*b* = 4.6181 (2) Å	µ = 0.08 mm^−^^1^
*c* = 37.3048 (15) Å	*T* = 293 K
*V* = 1967.63 (14) Å^3^	Prism, yellow
*Z* = 4	0.35 × 0.25 × 0.15 mm

### Data collection

**Table:**

Bruker Kappa APEXII diffractometer	1450 reflections with *I* > 2σ(*I*)
Radiation source: fine-focus sealed tube	*R*_int_ = 0.039
graphite	θ_max_ = 25.0°, θ_min_ = 3.3°
φ and ω scans	*h* = −13→12
9233 measured reflections	*k* = −5→5
1761 independent reflections	*l* = −43→44

### Refinement

**Table:**

Refinement on *F*^2^	Primary atom site location: structure-invariant direct methods
Least-squares matrix: full	Secondary atom site location: difference Fourier map
*R*[*F*^2^ > 2σ(*F*^2^)] = 0.038	Hydrogen site location: inferred from neighbouring sites
*wR*(*F*^2^) = 0.109	H-atom parameters constrained
*S* = 0.92	*w* = 1/[σ^2^(*F*_o_^2^) + (0.081*P*)^2^] where *P* = (*F*_o_^2^ + 2*F*_c_^2^)/3
1761 reflections	(Δ/σ)_max_ = 0.001
262 parameters	Δρ_max_ = 0.11 e Å^−^^3^
1 restraint	Δρ_min_ = −0.11 e Å^−^^3^

### Fractional atomic coordinates and isotropic or equivalent isotropic displacement parameters (Å^2^)

**Table:**

	*x*	*y*	*z*	*U*_iso_*/*U*_eq_	
N1	−0.0359 (3)	1.7957 (7)	0.50015 (10)	0.0616 (8)	
N2	0.1337 (2)	1.1963 (5)	0.43344 (7)	0.0397 (6)	
N3	0.4326 (3)	0.9705 (6)	0.20004 (8)	0.0532 (7)	
N4	0.7055 (3)	1.3287 (10)	0.11332 (10)	0.0816 (11)	
C1	0.1358 (3)	1.5338 (6)	0.48188 (8)	0.0375 (7)	
C2	0.1996 (3)	1.6715 (8)	0.50825 (9)	0.0522 (9)	
H2	0.2789	1.6316	0.5110	0.063*	
C3	0.1457 (4)	1.8681 (8)	0.53055 (9)	0.0576 (10)	
H3	0.1874	1.9615	0.5486	0.069*	
C4	0.0294 (4)	1.9221 (8)	0.52547 (10)	0.0577 (10)	
H4	−0.0067	2.0551	0.5406	0.069*	
C5	0.0193 (3)	1.6096 (8)	0.47908 (10)	0.0529 (9)	
H5	−0.0240	1.5229	0.4609	0.063*	
C6	0.1904 (3)	1.3248 (7)	0.45776 (9)	0.0429 (8)	
H6	0.2698	1.2849	0.4604	0.052*	
C7	0.1904 (3)	0.9962 (6)	0.41026 (8)	0.0357 (7)	
C8	0.3029 (3)	0.8895 (6)	0.41450 (8)	0.0409 (7)	
H8	0.3479	0.9497	0.4339	0.049*	
C9	0.3488 (3)	0.6942 (6)	0.39020 (9)	0.0415 (8)	
H9	0.4241	0.6231	0.3937	0.050*	
C10	0.2848 (3)	0.6020 (6)	0.36067 (8)	0.0370 (7)	
C11	0.1720 (3)	0.7078 (7)	0.35684 (9)	0.0440 (8)	
H11	0.1270	0.6491	0.3374	0.053*	
C12	0.1252 (3)	0.8988 (7)	0.38142 (9)	0.0416 (7)	
H12	0.0486	0.9630	0.3786	0.050*	
C13	0.3353 (3)	0.3988 (7)	0.33309 (9)	0.0459 (8)	
H13A	0.2798	0.2439	0.3287	0.055*	
H13B	0.4062	0.3125	0.3426	0.055*	
C14	0.3636 (3)	0.5460 (6)	0.29792 (9)	0.0419 (7)	
C15	0.4547 (3)	0.7423 (7)	0.29520 (9)	0.0452 (8)	
H15	0.4998	0.7826	0.3153	0.054*	
C16	0.4800 (3)	0.8798 (7)	0.26323 (9)	0.0469 (8)	
H16	0.5422	1.0092	0.2621	0.056*	
C17	0.4139 (3)	0.8275 (7)	0.23287 (9)	0.0480 (8)	
C18	0.3207 (3)	0.6355 (9)	0.23547 (10)	0.0614 (10)	
H18	0.2738	0.6004	0.2156	0.074*	
C19	0.2972 (3)	0.4961 (8)	0.26751 (10)	0.0572 (9)	
H19	0.2353	0.3656	0.2686	0.069*	
C20	0.5355 (4)	1.0079 (8)	0.18865 (9)	0.0565 (10)	
H20	0.5973	0.9275	0.2015	0.068*	
C21	0.5619 (3)	1.1718 (8)	0.15619 (9)	0.0517 (9)	
C22	0.6740 (3)	1.1852 (9)	0.14284 (10)	0.0683 (11)	
H22	0.7322	1.0870	0.1553	0.082*	
C23	0.6200 (4)	1.4715 (11)	0.09659 (12)	0.0764 (13)	
H23	0.6391	1.5737	0.0759	0.092*	
C24	0.5058 (4)	1.4774 (9)	0.10794 (11)	0.0704 (11)	
H24	0.4496	1.5825	0.0954	0.084*	
C25	0.4766 (3)	1.3259 (9)	0.13793 (11)	0.0607 (10)	
H25	0.3997	1.3257	0.1461	0.073*	

### Atomic displacement parameters (Å^2^)

**Table:**

	*U*^11^	*U*^22^	*U*^33^	*U*^12^	*U*^13^	*U*^23^
N1	0.0476 (17)	0.0680 (19)	0.069 (2)	0.0030 (15)	0.0021 (16)	−0.0211 (17)
N2	0.0379 (14)	0.0420 (14)	0.0392 (15)	0.0028 (11)	0.0002 (13)	−0.0020 (12)
N3	0.0526 (19)	0.0636 (18)	0.0434 (16)	0.0009 (15)	0.0023 (14)	0.0059 (14)
N4	0.059 (2)	0.125 (3)	0.061 (2)	−0.008 (2)	0.0122 (18)	0.011 (2)
C1	0.0387 (17)	0.0362 (15)	0.0375 (16)	−0.0039 (13)	0.0007 (14)	0.0037 (13)
C2	0.048 (2)	0.060 (2)	0.049 (2)	0.0018 (16)	−0.0086 (16)	−0.0053 (17)
C3	0.068 (3)	0.061 (2)	0.044 (2)	−0.0018 (19)	−0.0119 (19)	−0.0096 (17)
C4	0.066 (3)	0.054 (2)	0.053 (2)	0.0022 (19)	0.012 (2)	−0.0063 (17)
C5	0.041 (2)	0.059 (2)	0.058 (2)	−0.0039 (17)	−0.0033 (17)	−0.0148 (18)
C6	0.0354 (17)	0.0451 (16)	0.0483 (19)	0.0002 (14)	−0.0001 (15)	0.0008 (15)
C7	0.0358 (16)	0.0334 (13)	0.0379 (16)	−0.0009 (13)	0.0035 (13)	0.0041 (13)
C8	0.0374 (17)	0.0462 (16)	0.0390 (17)	−0.0015 (14)	−0.0040 (14)	0.0016 (15)
C9	0.0315 (16)	0.0404 (16)	0.053 (2)	0.0017 (13)	0.0014 (15)	0.0081 (15)
C10	0.0391 (17)	0.0310 (14)	0.0410 (18)	−0.0004 (12)	0.0051 (14)	0.0085 (13)
C11	0.0397 (17)	0.0465 (17)	0.0457 (19)	−0.0002 (14)	−0.0029 (15)	−0.0093 (15)
C12	0.0320 (16)	0.0445 (16)	0.0481 (19)	0.0041 (13)	−0.0017 (15)	−0.0021 (15)
C13	0.0472 (19)	0.0360 (15)	0.055 (2)	0.0048 (14)	0.0089 (16)	0.0036 (15)
C14	0.0427 (18)	0.0372 (15)	0.0459 (19)	0.0064 (14)	0.0083 (16)	−0.0029 (15)
C15	0.0485 (19)	0.0443 (16)	0.0427 (18)	0.0014 (14)	−0.0003 (15)	−0.0013 (15)
C16	0.046 (2)	0.0480 (17)	0.0469 (19)	−0.0036 (15)	0.0033 (17)	−0.0009 (15)
C17	0.046 (2)	0.052 (2)	0.0457 (19)	0.0036 (16)	0.0028 (17)	0.0021 (15)
C18	0.057 (2)	0.081 (3)	0.046 (2)	−0.008 (2)	−0.0074 (18)	0.002 (2)
C19	0.050 (2)	0.059 (2)	0.062 (2)	−0.0119 (17)	0.0007 (17)	0.0002 (18)
C20	0.058 (2)	0.065 (2)	0.047 (2)	0.0088 (19)	0.0000 (17)	0.0021 (18)
C21	0.051 (2)	0.062 (2)	0.042 (2)	0.0009 (17)	−0.0007 (17)	−0.0020 (17)
C22	0.054 (2)	0.100 (3)	0.050 (2)	0.003 (2)	0.0016 (19)	0.005 (2)
C23	0.086 (3)	0.096 (3)	0.047 (2)	−0.015 (3)	0.009 (2)	0.010 (2)
C24	0.074 (3)	0.082 (3)	0.055 (2)	0.003 (2)	0.002 (2)	0.016 (2)
C25	0.053 (2)	0.080 (2)	0.048 (2)	−0.0018 (19)	0.0048 (19)	0.0078 (18)

### Geometric parameters (Å, °)

**Table:**

N1—C5	1.325 (5)	C11—C12	1.380 (4)
N1—C4	1.338 (5)	C11—H11	0.9300
N2—C6	1.263 (4)	C12—H12	0.9300
N2—C7	1.422 (4)	C13—C14	1.513 (4)
N3—C20	1.262 (5)	C13—H13A	0.9700
N3—C17	1.408 (4)	C13—H13B	0.9700
N4—C23	1.334 (6)	C14—C15	1.384 (4)
N4—C22	1.335 (5)	C14—C19	1.384 (5)
C1—C5	1.380 (5)	C15—C16	1.382 (5)
C1—C2	1.379 (5)	C15—H15	0.9300
C1—C6	1.459 (4)	C16—C17	1.382 (5)
C2—C3	1.377 (5)	C16—H16	0.9300
C2—H2	0.9300	C17—C18	1.388 (5)
C3—C4	1.364 (5)	C18—C19	1.384 (5)
C3—H3	0.9300	C18—H18	0.9300
C4—H4	0.9300	C19—H19	0.9300
C5—H5	0.9300	C20—C21	1.459 (5)
C6—H6	0.9300	C20—H20	0.9300
C7—C8	1.385 (4)	C21—C22	1.375 (5)
C7—C12	1.384 (4)	C21—C25	1.386 (5)
C8—C9	1.382 (4)	C22—H22	0.9300
C8—H8	0.9300	C23—C24	1.372 (6)
C9—C10	1.389 (4)	C23—H23	0.9300
C9—H9	0.9300	C24—C25	1.361 (5)
C10—C11	1.386 (4)	C24—H24	0.9300
C10—C13	1.507 (4)	C25—H25	0.9300
			
C5—N1—C4	115.9 (3)	C10—C13—H13A	108.9
C6—N2—C7	120.6 (3)	C14—C13—H13A	108.9
C20—N3—C17	119.9 (3)	C10—C13—H13B	108.9
C23—N4—C22	115.7 (4)	C14—C13—H13B	108.9
C5—C1—C2	116.5 (3)	H13A—C13—H13B	107.8
C5—C1—C6	122.2 (3)	C15—C14—C19	117.4 (3)
C2—C1—C6	121.3 (3)	C15—C14—C13	121.2 (3)
C3—C2—C1	120.0 (3)	C19—C14—C13	121.3 (3)
C3—C2—H2	120.0	C16—C15—C14	121.4 (3)
C1—C2—H2	120.0	C16—C15—H15	119.3
C4—C3—C2	118.1 (3)	C14—C15—H15	119.3
C4—C3—H3	120.9	C15—C16—C17	120.9 (3)
C2—C3—H3	120.9	C15—C16—H16	119.6
N1—C4—C3	124.2 (3)	C17—C16—H16	119.6
N1—C4—H4	117.9	C16—C17—C18	118.2 (3)
C3—C4—H4	117.9	C16—C17—N3	123.2 (3)
N1—C5—C1	125.4 (3)	C18—C17—N3	118.5 (3)
N1—C5—H5	117.3	C19—C18—C17	120.4 (3)
C1—C5—H5	117.3	C19—C18—H18	119.8
N2—C6—C1	122.3 (3)	C17—C18—H18	119.8
N2—C6—H6	118.8	C18—C19—C14	121.6 (3)
C1—C6—H6	118.8	C18—C19—H19	119.2
C8—C7—C12	118.2 (3)	C14—C19—H19	119.2
C8—C7—N2	125.7 (3)	N3—C20—C21	122.9 (3)
C12—C7—N2	116.1 (3)	N3—C20—H20	118.6
C9—C8—C7	120.6 (3)	C21—C20—H20	118.6
C9—C8—H8	119.7	C22—C21—C25	117.0 (3)
C7—C8—H8	119.7	C22—C21—C20	121.1 (4)
C8—C9—C10	121.4 (3)	C25—C21—C20	121.9 (3)
C8—C9—H9	119.3	N4—C22—C21	124.9 (4)
C10—C9—H9	119.3	N4—C22—H22	117.5
C11—C10—C9	117.6 (3)	C21—C22—H22	117.5
C11—C10—C13	120.3 (3)	N4—C23—C24	124.2 (4)
C9—C10—C13	122.1 (3)	N4—C23—H23	117.9
C12—C11—C10	121.1 (3)	C24—C23—H23	117.9
C12—C11—H11	119.4	C25—C24—C23	118.4 (4)
C10—C11—H11	119.4	C25—C24—H24	120.8
C11—C12—C7	121.1 (3)	C23—C24—H24	120.8
C11—C12—H12	119.5	C24—C25—C21	119.7 (4)
C7—C12—H12	119.5	C24—C25—H25	120.1
C10—C13—C14	113.2 (2)	C21—C25—H25	120.1
			
C5—C1—C2—C3	1.4 (5)	C10—C13—C14—C15	69.5 (4)
C6—C1—C2—C3	179.9 (3)	C10—C13—C14—C19	−108.5 (3)
C1—C2—C3—C4	−0.5 (5)	C19—C14—C15—C16	−1.1 (5)
C5—N1—C4—C3	−0.5 (6)	C13—C14—C15—C16	−179.2 (3)
C2—C3—C4—N1	0.1 (6)	C14—C15—C16—C17	0.7 (5)
C4—N1—C5—C1	1.6 (6)	C15—C16—C17—C18	0.6 (5)
C2—C1—C5—N1	−2.0 (5)	C15—C16—C17—N3	177.2 (3)
C6—C1—C5—N1	179.5 (3)	C20—N3—C17—C16	43.1 (5)
C7—N2—C6—C1	179.4 (3)	C20—N3—C17—C18	−140.4 (4)
C5—C1—C6—N2	−1.6 (5)	C16—C17—C18—C19	−1.5 (5)
C2—C1—C6—N2	180.0 (3)	N3—C17—C18—C19	−178.3 (3)
C6—N2—C7—C8	10.1 (4)	C17—C18—C19—C14	1.1 (6)
C6—N2—C7—C12	−171.1 (3)	C15—C14—C19—C18	0.2 (5)
C12—C7—C8—C9	0.9 (4)	C13—C14—C19—C18	178.3 (3)
N2—C7—C8—C9	179.8 (3)	C17—N3—C20—C21	−175.5 (3)
C7—C8—C9—C10	0.8 (4)	N3—C20—C21—C22	−173.9 (4)
C8—C9—C10—C11	−1.4 (4)	N3—C20—C21—C25	7.6 (6)
C8—C9—C10—C13	177.4 (3)	C23—N4—C22—C21	1.3 (7)
C9—C10—C11—C12	0.3 (4)	C25—C21—C22—N4	−1.8 (6)
C13—C10—C11—C12	−178.6 (3)	C20—C21—C22—N4	179.7 (4)
C10—C11—C12—C7	1.5 (5)	C22—N4—C23—C24	0.0 (7)
C8—C7—C12—C11	−2.1 (4)	N4—C23—C24—C25	−0.8 (7)
N2—C7—C12—C11	178.9 (3)	C23—C24—C25—C21	0.2 (6)
C11—C10—C13—C14	71.8 (4)	C22—C21—C25—C24	0.9 (5)
C9—C10—C13—C14	−107.1 (3)	C20—C21—C25—C24	179.4 (4)
